# Medical Boards of Georgia Abolished

**Published:** 1881-09-20

**Authors:** T. S. Powell


### *MEDICAL BOARDS OF GEORGIA ABOLISHED.*

At the late session of the Georgia Legislature, a bill purporting to come from the Georgia Medical Association, was introduced to abolish the Allopathic, Eclectic and Thompsonian Medical Boards of the State, and to establish a new board composed of nine physicians, appointed by the Governor, who were to have authority to examine and license all applicants to practice in any and all of the different systems and isms.

While it affords me no pleasure to oppose any action of the State Medical Society, and advocated by certain medical friends in my own city, yet I was compelled to do so, because it contained a feature which was in direct conflict with the ethics as announced by a resolution of the American Medical Association at its late meeting, which body I attended as a representative of the profession of Georgia.

The action of the American Medical Association to which I refer, is in the following language:

"It is not in accord with the interest of the public or the honor of the profession that any physician or medical teacher should examine or sign diplomas or certificates of proficiency for, or otherwise be specially concerned with, the graduation of persons whom they have good reason to believe intend to support and practice any exclusive and irregular system of medicine."

This unprofessional feature of the bill, with other objections, I explained to the committee, and the bill was abandoned, and a second bill was framed and presented.

In justice to the profession and the members of the old Board I felt also constrained to oppose this bill, and did so in a circular embracing the following objections:

It appears to be actuated by the same determination that characterized its predecessors, and that is *the deposition of the members of the present Board.*

It discriminates against the graduates outside of the State. In other words, a graduate of any medical institution in the State of Georgia who registers may practice medicine, independent of the Board, whether he be a man of good or bad character; but a graduate of another State must not only pay a fee of $20, but is subject to the judgment of the Board in respect to his moral character, and "such other of his qualifications as said Board may require."

Again, there is doubt as to whether or not physicians of long practice and high professional standing, may not be liable to pay a fee of $20 to secure the endorsement of their diplomas, the section on this point being in uncertain language, or admitting, at least, of different constructions.

This second bill, like the first, was likewise abandoned, and a third one adopted abolishing all Boards, and if signed by the Governor, will be the law of the State. We publish it in the present issue of our Journal.

While I favor the requirements of this bill, and think it would accomplish great good if it could be enforced, yet had I known of this bill in time I would have proposed an amendment extending the license of those practicing under temporary license, one or two years, that they might have time to prepare for college and to procure diplomas in the regular way.

There is another feature in the bill which I need not now mention, that I would have suggested some alteration in upon the ground of its probable unconstitutionality, and which may defeat the entire objects of the bill, if tested in the courts.

Thus much I have felt called upon to say in justice to myself in connection with the discussion upon this bill. In all my action upon this subject I have openly avowed my objects and purposes, and have never lost sight of the interest and honor of the Medical Profession, and desire my views and motives to be fully known and understood by the profession everywhere.

T. S. POWELL.

